# Integrated miRNA–mRNA Profiling of C2C12 Myoblasts Indicates Regulatory Interactions Involved in Proliferation and Differentiation

**DOI:** 10.3390/biology14050574

**Published:** 2025-05-20

**Authors:** Xiaolong Chang, Junwu Ma

**Affiliations:** National Key Laboratory for Swine Genetic Improvement and Germplasm Innovation, Jiangxi Agricultural University, Nanchang 330045, China

**Keywords:** C2C12 myoblasts, miRNA, proliferation, differentiation, regulatory network

## Abstract

Myogenesis is a complex biological process regulated by multiple factors. This study systematically revealed the dynamic changes of gene expression and its regulatory network in C2C12 myoblasts during proliferation and differentiation stages by integrating transcriptome and miRNA-omics data. The analysis results showed that in the early stage of proliferation, gene expression showed significant fluctuations, and key cell cycle and DNA replication-related genes were closely associated with specific miRNAs (miR-486a-5p, miR-486b-5p, and miR-351-5p), suggesting that these miRNAs play an important role in regulating cell proliferation. In the differentiation stage, the activation of key myogenic transcription factors and signaling pathways, such as MAPK and PI3K-Akt, synergizes with miRNA regulation to promote the myogenic program. In addition, we found that genes such as *IGF1* and *Dio2* were continuously upregulated during differentiation, and IGF1 might be regulated by multiple miRNAs during this process. This study provides key molecular insights for a deeper understanding of muscle development and regeneration.

## Simple Summary

This study used a combination of transcriptomics and microRNA genomics to reveal the dynamic changes in gene expression and its regulatory network during the proliferation and differentiation of C2C12 myoblasts. It provided important molecular mechanism insights for a deeper understanding of muscle development and regeneration.

## 1. Introduction

Skeletal muscle accounts for approximately 40% of the human body weight and plays a crucial role in locomotion, metabolic regulation, and energy homeostasis [1,2]. Myogenesis, the process through which myoblasts proliferate, differentiate, and fuse to form functional muscle fibers, is a highly orchestrated biological event. This intricate process is meticulously governed by a multifaceted regulatory network that encompasses transcription factors, epigenetic modifications, and non-coding RNAs [3,4,5]. Notably, microRNA (miRNA), a subset of small non-coding RNAs [6], serves as a crucial regulator in muscle development. It executes its regulatory function by targeting the mRNAs of protein-coding genes (PCGs) for either degradation or translational inhibition [7].

The C2C12 cell line, derived from immortalized mouse myoblasts, is a widely recognized model for simulating in vitro myogenesis [8,9,10]. During the proliferation stage, these cells remain undifferentiated. However, when exposed to conditions of reduced serum concentration, they swiftly exit the cell cycle and begin differentiation, ultimately fusing into multinucleated myotubes. This process is marked by the spatiotemporal regulation of muscle-specific genes such as *Myod1*, *Myf5*, *MEF2*, and myosin heavy chain (MHC) [11,12].

In recent years, the advent of high-throughput sequencing technology has facilitated the comprehensive analysis of gene and non-coding RNA expression profiles [13,14]. This has unveiled new opportunities for understanding the molecular regulatory mechanisms underlying muscle development [15,16]. Research has pinpointed certain miRNAs, notably muscle-specific miRNAs (myomiRs), including miR-1, miR-133, and miR-206, as instrumental in muscle proliferation and differentiation through the regulation of various target genes [17,18]. Specifically, miR-1 and miR-206 are inclined to suppress cell proliferation, favoring differentiation, whereas miR-133 predominantly stimulates myogenic cell proliferation [19,20]. While some studies have elucidated the roles of certain miRNAs in muscle development, a comprehensive understanding of the transcriptomic and miRNA dynamics throughout the transition of C2C12 cells from proliferation to differentiation, along with their interaction network, remains elusive.

In this study, we employed high-throughput sequencing technology to conduct a systematic analysis of the transcriptome and miRNA expression profiles of C2C12 cells during the proliferation stage (0 h, 12 h, 24 h, 36 h, 48 h, 60 h, 72 h) and differentiation stage (D0, D1, D3, D5, D8). Utilizing weighted gene co-expression network analysis (WGCNA) and pathway enrichment analysis, we identified key regulatory modules and signaling pathways intimately associated with myoblast proliferation and differentiation. The constructed mRNA-miRNA interaction network revealed the potential regulatory mechanism of miRNA in the process of myogenesis. Notably, we discovered that miR-486a-5p, miR-486b-5p, and miR-351-5p may regulate the early proliferation of myoblasts by targeting multiple key genes. Furthermore, genes such as *IGF1* demonstrated sustained high expression at all stages of differentiation and were regulated by distinct miRNAs. In conclusion, this study not only offers new insights into the molecular mechanisms underlying skeletal muscle development but also establishes a foundation for potential intervention strategies for muscle-related diseases and regenerative medicine.

## 2. Materials and Methods

### 2.1. Cell Culture and Differentiation Induction

C2C12 cell line (SCSP-505) was purchased from the Cell Bank of the Chinese Academy of Sciences (Shanghai, China). The cells used in the experiment were maintained at a low passage number (P5–P10), seeded in a 6-well plate at a density of 5 × 10^4^ cells/well, and cultured in growth medium (GM) containing 10% fetal bovine serum and 1% penicillin–streptomycin [21]. When the confluence of C2C12 cells reached 80% to 90%, the GM was replaced with differentiation medium (DM) containing 2% horse serum (Gibco, Auckland, New Zealand) to induce myogenic differentiation. All cells were cultured in a humidified incubator at 37 °C and 5% CO_2_.

### 2.2. Cell Proliferation Assay

Cell proliferation was measured at 0, 12, 24, 36, and 48 h using the Cell Counting Kit 8 (CCK-8; Dojindo, Japan) according to the manufacturer’s instructions.

### 2.3. RNA Extraction and Library Preparation

Total RNA was extracted from C2C12 cells using TRIzol reagent (15,596,026, Invitrogen, Carlsbad, CA, USA) according to the manufacturer’s instructions. The quality and integrity of the extracted RNA were verified using standard methods, including NanoDrop spectrophotometry and agarose gel electrophoresis. Subsequently, the cDNA library was constructed from the purified RNA samples and sequenced by BGI (Shenzhen, China). The constructed library was sequenced on the MGISEQ-2000 system to generate 150 bp paired-end sequencing sequences, and the small RNA library was deeply sequenced on the DNBSEQ-SE50 platform.

### 2.4. RNA-Seq Analysis

FastQC was initially utilized for quality control of sequencing data. HISAT2 (v2.2.0) [22] and featureCounts (v2.0.1) [23] software were employed for read alignment and gene expression quantification, respectively. Differential expression analysis was conducted using the DESeq2 (v1.42.1) package in R (v4.3.1). Differentially expressed genes (DEGs) between the two groups were identified based on the following criteria: q-value (adjusted *p*) < 0.01 and fold change > 2. Functional enrichment analysis of the DEGs was performed using the R package clusterProfiler (v4.10.1) [24] to explore their biological significance.

### 2.5. Small RNA Sequencing Analysis

FastQC was initially utilized for quality control of sequencing data. Then, miRDeep2 (v0.1.3) [25] software was used for analysis and the quality-controlled reads were aligned to the mouse miRNA database (miRBase) using the mapper.pl module, quantified using the quantifier.pl module. Differential expression analysis was conducted using the DESeq2 package in R. Differentially expressed miRNA (DE-miRNA) between the two groups was identified based on the following criteria: q-value (adjusted *p*) < 0.01 and fold change > 2.

### 2.6. Time Series and WGCNA Analysis

The Mfuzz [26] software (v2.66.0) was utilized for time series analysis, while WGCNA (v1.73) [27] was employed for gene co-expression network analysis.

### 2.7. Construction of mRNA-miRNA Regulatory Network

The R package multiMiR (v1.24.0) [28] was used to predict differentially expressed miRNA target genes. The predicted target genes were crossed with the differential mRNA data to analyze the miRNA–mRNA pairs with negative regulatory relationships. The miRNA–mRNA targeting relationship network was drawn using Cytoscape (v3.10.3) [29] software.

### 2.8. Statistical Analysis

All data were acquired from a minimum of three experiments, analyzed using R 4.3.1 software (GraphPad Software, San Diego, CA, USA), and are presented as mean ± standard deviation (SD). *p* < 0.05 was considered statistically significant. Throughout the paper, the significance level is indicated as follows: ns (not significant), * *p* ≤ 0.05, ** *p* ≤ 0.01, *** *p* ≤ 0.001.

## 3. Results

### 3.1. Transcriptome Dynamics During Proliferation and Differentiation of C2C12 Myoblasts

**Figure 1:**
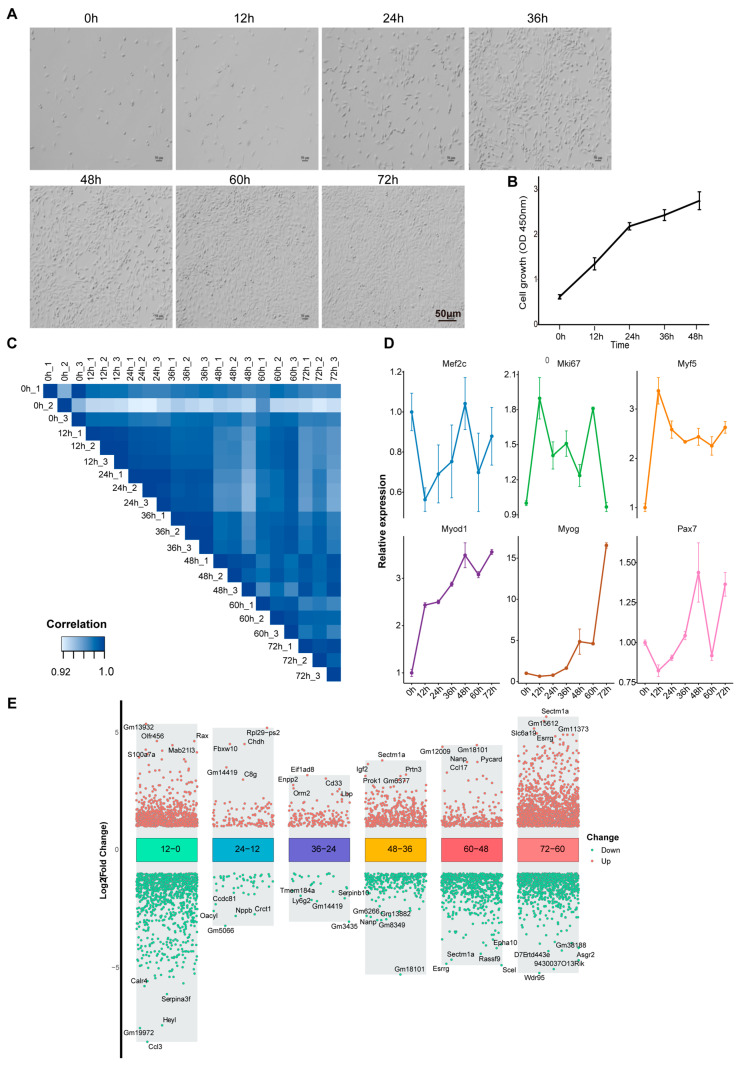
Dynamic changes of phenotype and transcriptome during proliferation of C2C12 cells. (**A**) Phenotype of C2C12 cells during proliferation (0–72 h). (**B**) CCK8 assay of cell proliferation in C2C12 cells (*n* = 5). Graph bars represent mean ± SEM. (**C**) Sample correlation heatmap. (**D**) RNA-seq analysis of myogenic gene expression during C2C12 cell proliferation (*n* = 3). Graph bars represent mean ± SEM. (**E**) Volcano plot of differentially expressed genes at adjacent time points.

The classical model of C2C12 myoblasts is largely used in muscle development studies. We first characterized the morphological changes and proliferation dynamics of C2C12 cells during proliferation (0–72 h). We observed a significant increase in cell number over the culture period (Figure S1), reaching nearly 100% confluence by 60 h (Figure 1A). Through CCK-8 cell proliferation analysis, we found that the cells showed a high proliferation rate in the first 24 h, while the proliferation rate slowed down relatively between 24–48 h (Figure 1B), indicating that cell contact inhibition may begin to play a role at this stage.

In order to comprehensively analyze the gene expression dynamics during C2C12 cell proliferation and differentiation, we performed high-throughput RNA sequencing on cells in the proliferation stage (0 h, 12 h, 24 h, 36 h, 48 h, 60 h, 72 h) and differentiation stage (D0, D1, D3, D5, D8). The Pearson correlation coefficients between samples were calculated based on gene expression level data and were all greater than 0.8, ensuring high quality and reliability of the data (Figure 1C). We paid special attention to the expression patterns of classic genes related to muscle development, including *Pax7*, *Myf5*, *Myod1*, *Myog*, *Mef2c*, and the proliferation marker *Mki67*. These genes showed significant oscillatory expression patterns during proliferation (Figure 1D), suggesting the complexity of transcriptional regulation during cell proliferation. Analysis of differentially expressed genes (DEGs) showed that the amplitude of changes in gene expression levels between different adjacent time points showed obvious dynamic characteristics (Figure 1E). Among them, the difference between 12 h and 0 h was the most significant, especially in genes closely related to cell proliferation, such as *Ccne1*, *Ccne2*, and *Mcm2*, which were significantly upregulated at 12 h. This result suggests that key transcriptional reprogramming events may have occurred in the early stages of cell proliferation.

**Figure 2:**
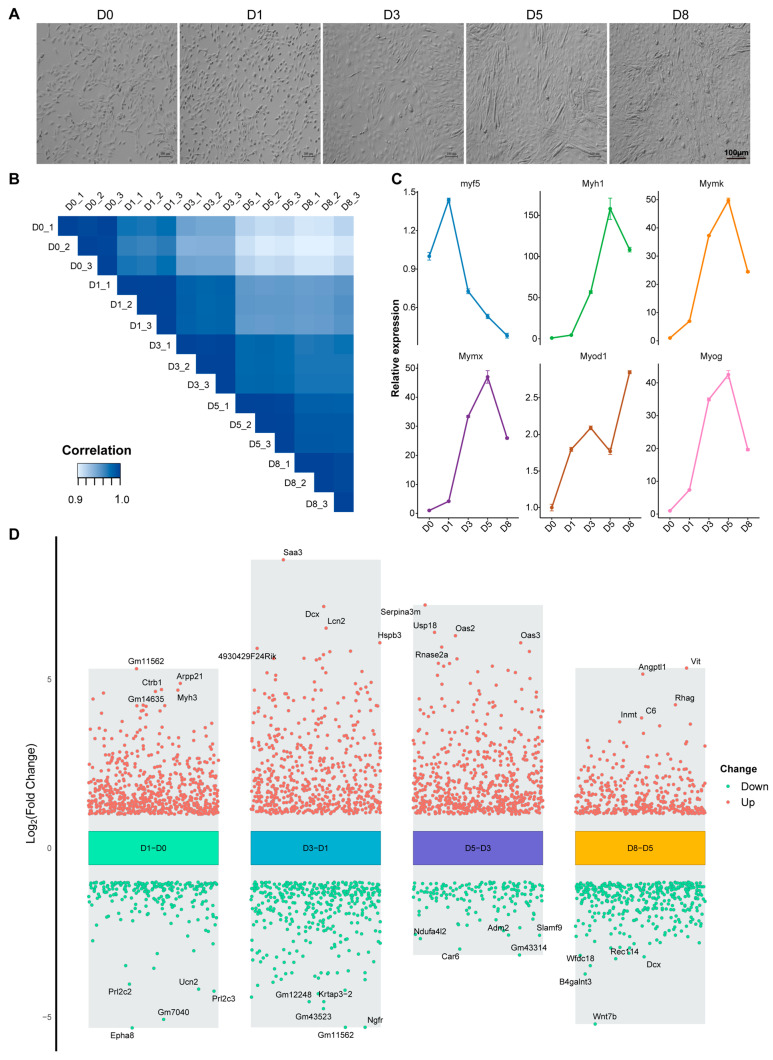
Dynamic changes of phenotype and transcriptome during differentiation of C2C12 cells. (**A**) Phenotype of C2C12 cells during differentiation (D0–D8). (**B**) Sample correlation heatmap. (**C**) RNA-seq analysis of myogenic gene expression during C2C12 cell differentiation (*n* = 3). Graph bars represent mean ± SEM. (**D**) Volcano plot of differentially expressed genes at adjacent time points.

During differentiation, C2C12 cells gradually fused to form multinucleated myotubes, and typical myotube structures could be observed at D3 (Figure 2A). The transcriptome data of the differentiation process also showed a high correlation (Figure 2B). Key regulatory factors for muscle development showed a clear temporal expression pattern: *Myf5* expression was gradually downregulated from D1, while *Myod1*, *Myog*, and myotube fusion essential factors *Mymk*, *Mymx*, and myosin heavy chain *Myh1* showed an upregulation trend during differentiation (Figure 2C). Analysis of differentially expressed genes between adjacent differentiation time points showed that gene expression remodeling mainly occurred in the early stages of differentiation (Figure 2D). Through further analysis, we identified a group of genes that were continuously upregulated during differentiation (Figure S2A), including *Dio2*, *IGF1*, *Lum*, *Fxyd1*, *Pappa2*, and *Amy1* (Figure S2B), which may play key regulatory roles in myogenic differentiation.

### 3.2. Co-Expression Analysis of mRNAs During Proliferation and Differentiation of C2C12 Myoblasts

**Figure 3:**
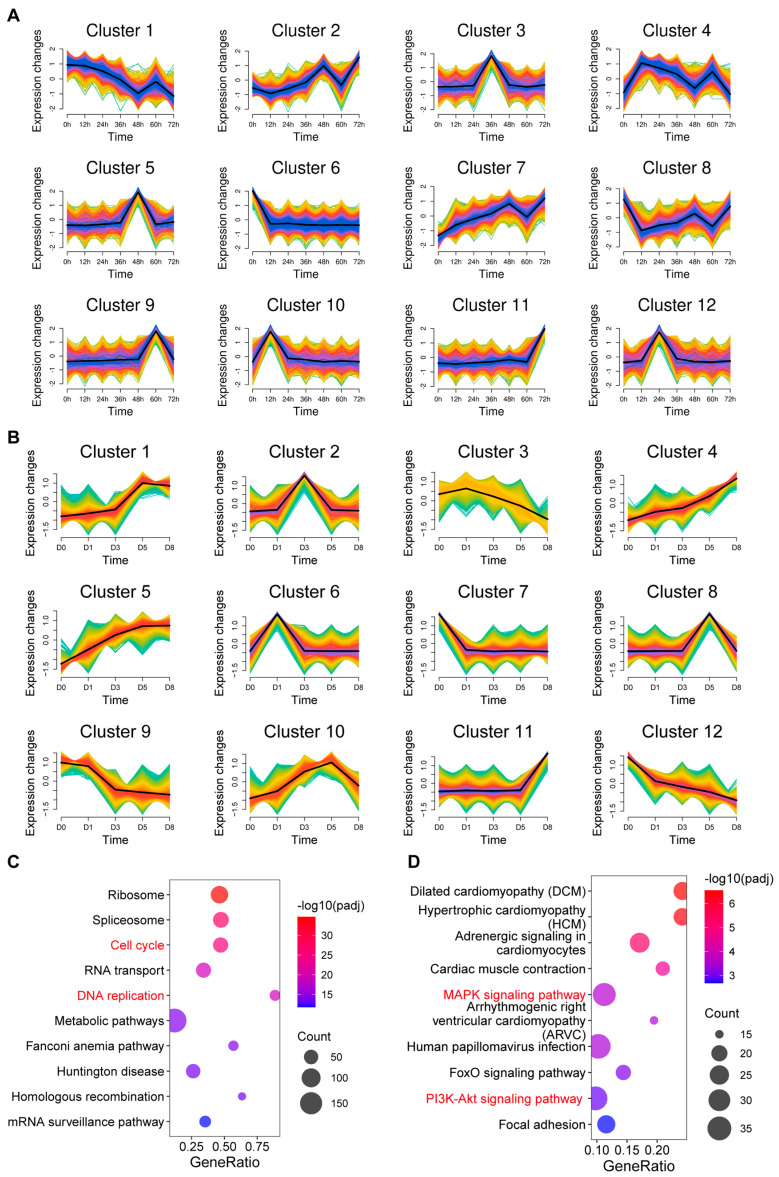
Co-expression analysis of mRNA during proliferation and differentiation of C2C12 cells. (**A**) Fuzzy c-means clustering identified 12 distinct temporal patterns of gene expression. The *x*-axis represents the seven proliferation stages, while the *y*-axis represents the log2-transformed, normalized intensity ratio of each stage. (**B**) Fuzzy c-means clustering identified 12 distinct temporal patterns of gene expression. The *x*-axis represents the five differentiation stages, while the *y*-axis represents the log2-transformed, normalized intensity ratio of each stage. (**C**) Kegg enrichment analysis of cluster 4 genes in (**A**). (**D**) Kegg enrichment analysis of cluster 10 genes in (**B**).

Genes with similar expression patterns often participate in related biological functions and pathways [30,31]. To identify key regulatory gene modules, we performed unsupervised clustering of the gene expression patterns of proliferation and differentiation processes into 12 expression clusters for each process using Mfuzz software (v2.66.0) (Figure 3A,B).

During proliferation, muscle progenitor cell markers *Pax3* and *Pax7* were mainly enriched in cluster 2, while key cell cycle regulators (*Ccne1*, *Ccne2*, *Ccna2*, *PCNA*, and *Mki67*) were enriched in cluster 4. Interestingly, key genes regulating muscle differentiation *(Myog*, *Myh1*, *Myh2*, *Mymx*, and *Mymk*) were mainly enriched in cluster 11. KEGG pathway analysis of genes in cluster 4 showed that these genes were significantly enriched in cell cycle and DNA replication-related pathways (Figure 3C), further confirming that this gene module has an important regulatory role in cell proliferation.

During differentiation, myosin heavy chain genes (*Myh1*, *Myh2*) were mainly enriched in cluster 1, while key regulatory factors for myogenic differentiation (*Myog*, *Mymx*, and *Mymk*) were enriched in cluster 10. A KEGG pathway analysis of cluster 10 genes revealed that these genes were significantly enriched in signaling pathways such as MAPK and PI3K-Akt (Figure 3D), suggesting that these signaling pathways play an important regulatory role in muscle differentiation.

### 3.3. Identification of Key Co-Expression Modules Based on WGCNA

**Figure 4:**
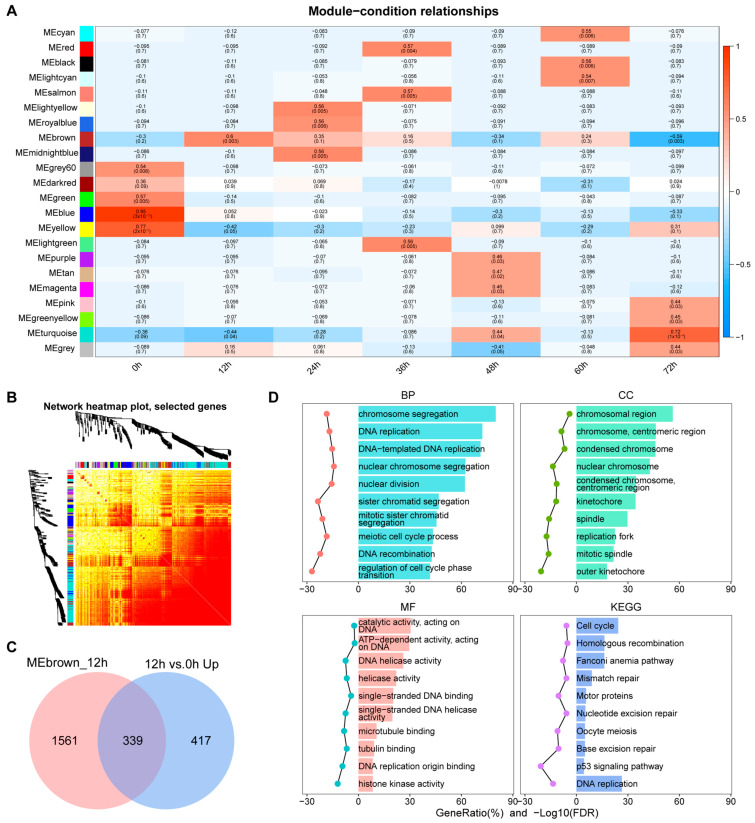
WGCNA analysis of C2C12 cell proliferation stages. (**A**) Heat map of correlation between gene modules and time groups. (**B**) Heat map of gene co-expression network. (**C**) Venn diagram of 12 h candidate modules and differentially upregulated genes at 12 h vs. 0 h. (**D**) GO and KEGG enrichment analysis of 339 key genes in (**C**).

In order to systematically identify the co-expressed gene modules and potential core regulatory genes, we performed weighted gene co-expression network analysis (WGCNA) on the transcriptome data. Figure 4A shows the results of WGCNA analysis in the proliferation stage. Figure 4B shows the corresponding gene co-expression network heat map. We found that the genes at the 12 h time point were significantly enriched in the brown module. Further through module attribution and gene significance analysis (Figure S3A), we screened out genes with high connectivity (MM ≥ 0.8) and high significance (GS ≥ 0.2) and took the intersection with the up-regulated genes at 12 h vs. 0 h and obtained 339 important genes (Figure 4C). These genes were significantly enriched in proliferation-related pathways such as DNA replication and cell cycle in functional enrichment analysis (Figure 4D), indicating that they play a core role in transcriptional activation in the early stage of proliferation.

**Figure 5:**
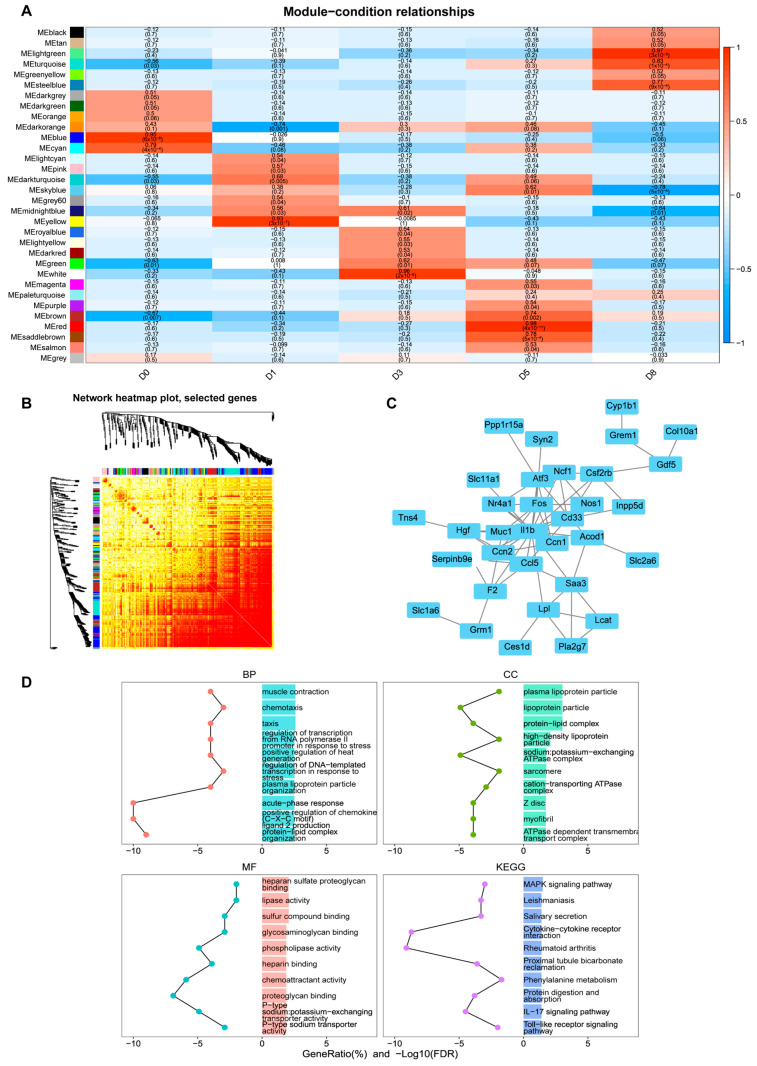
WGCNA analysis of C2C12 cell differentiation stages. (**A**) Heat map of correlation between gene modules and time groups. (**B**) Heat map of gene co-expression network. (**C**) PPI analysis of the intersection genes between the candidate module and significantly up-regulated genes at adjacent time points. (**D**) GO and KEGG enrichment analysis of key genes in (**C**).

Similarly, we performed WGCNA analysis on the differentiation stage and found that genes at different differentiation time points were significantly enriched in specific modules: D1 in the yellow module, D3 in the white module, D5 in the red module, and D8 in the green module (Figure 5A and Figure S3B). Figure 5B shows the heat map of the gene co-expression network in the differentiation stage. Through the intersection analysis of each significant enrichment module and the significantly up-regulated genes at adjacent time points, combined with protein interaction network (PPI) analysis (Figure 5C), we found that these genes were significantly enriched in differentiation regulation-related signaling pathways such as MAPK (Figure 5D), indicating that they play a key regulatory role in the muscle differentiation program.

### 3.4. Expression Patterns of miRNAs During Proliferation and Differentiation of C2C12 Myoblasts

**Figure 6:**
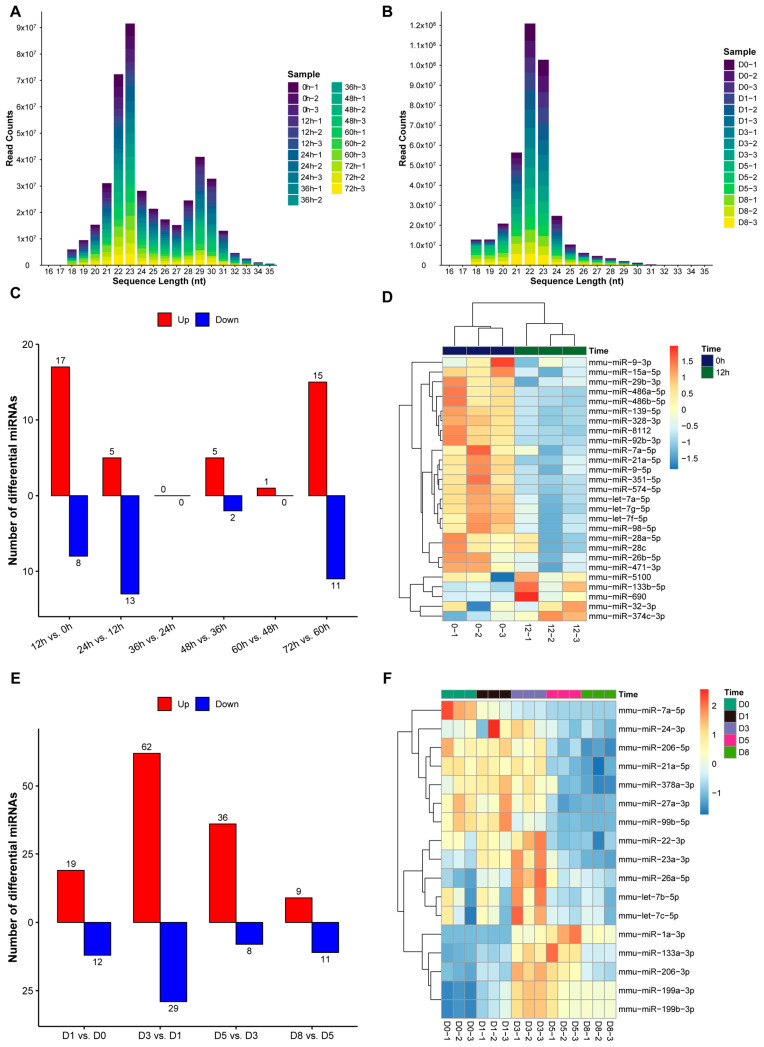
miRNA expression profiles during proliferation and differentiation of C2C12 cells. (**A**) Distribution of small RNA sequence lengths in proliferation samples. (**B**) Distribution of small RNA sequence lengths in differentiation samples. (**C**) Histogram of differential miRNAs at adjacent time points of proliferation. (**D**) Heat map of DE miRNAs between 12 h and 0 h of proliferation. (**E**) Histogram of differential miRNAs at adjacent time points of differentiation. (**F**) Heat map of DE miRNAs between differentiation time points.

To explore the regulatory role of noncoding RNA in muscle development, we performed miRNA sequencing in the proliferation and differentiation stages of C2C12 cells. The miRNAs obtained by sequencing were mainly concentrated in the length range of 21–23 nt (Figure 6A,B), which is consistent with the typical characteristics of mature miRNAs. Differential expression analysis showed that there were miRNAs with significant expression changes at adjacent time points in the proliferation stage (Figure 6C). It is worth noting that miR-133b-5p, miR-690, and miR-374c-3p were significantly upregulated at 12 h (Figure 6D), suggesting that they may play an important regulatory function in the early stage of proliferation.

The expression of miRNAs in the differentiation stage also showed significant dynamic changes (Figure 6E). Muscle-specific miRNA family members, including miR-1a-3p, miR-133a-3p, and miR-206-3p, gradually increased in expression during differentiation (Figure 6F), which is consistent with the function of these miRNAs in promoting muscle differentiation reported in previous studies [32,33]. In contrast, miR-7a-5p was significantly downregulated during differentiation, suggesting that it may play a role in maintaining the proliferative state of muscle precursor cells.

### 3.5. Target Gene Prediction and Functional Analysis of Differentially Expressed miRNAs

To reveal the regulatory network of miRNA, we predicted the target genes of differentially expressed miRNAs and integrated the differentially expressed gene data for analysis. In the proliferation stage, there were 220 overlapping genes between the predicted target genes of up-regulated miRNAs and down-regulated DEGs at 12 h vs. 0 h, while there were 81 overlapping genes between the predicted target genes of down-regulated miRNAs and up-regulated DEGs (Figure S4), indicating that these miRNA–mRNA pairs may have a negative regulatory relationship. There was also a significant miRNA–mRNA complementary regulation phenomenon at other time points (Figure S4). Functional enrichment analysis showed that these genes regulated by miRNAs were mainly involved in biological processes such as the cell cycle and were enriched in signaling pathways such as PI3K-Akt (Figure S5A).

During differentiation, the predicted target genes of up-regulated miRNAs and down-regulated DEGs, as well as the predicted target genes of down-regulated miRNAs and up-regulated DEGs, showed significant overlap between adjacent time points (Figure S4). These miRNA-regulated genes were significantly enriched in biological processes such as myogenic differentiation in GO analysis, while KEGG analysis showed that they were enriched in signaling pathways such as MAPK (Figure S5B), further confirming the key regulatory role of miRNA in muscle differentiation.

### 3.6. The miRNA–mRNA Interaction Network Reveals the Key Regulatory Axes of Muscle Development

**Figure 7:**
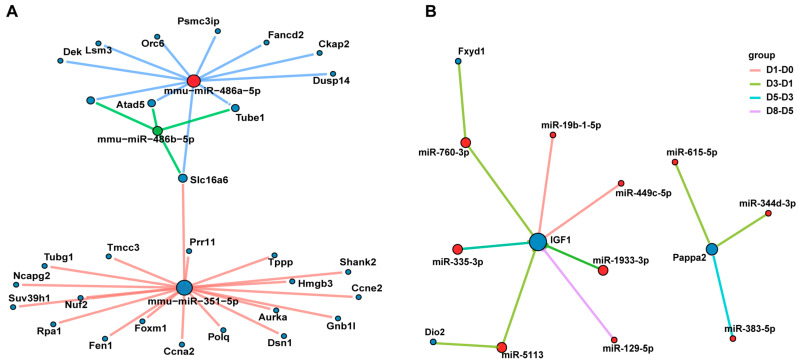
miRNA–mRNA interaction analysis. (**A**) Interaction network of miRNAs and genes significantly enriched at 12 h of proliferation. (**B**) Interaction network of genes that are continuously upregulated during differentiation and negatively regulated miRNAs.

To identify the miRNA regulatory network in the key stage of proliferation, we cross-analyzed the 339 key genes upregulated at 12 h vs. 0 h and the target genes negatively regulated by miRNAs, and found 29 overlapping genes, which were mainly targeted and regulated by miR-486a-5p, miR-486b-5p, and miR-351-5p (Figure 7A), suggesting that these miRNAs may regulate cell proliferation by inhibiting the expression of proliferation-related genes.

During differentiation, we identified six key genes (*Dio2*, *IGF1*, *Lum*, *Fxyd1*, *Pappa2*, and *Amy1*) that were consistently upregulated and further analyzed potential miRNAs that may regulate the expression of these genes. By constructing a miRNA–mRNA interaction network, we observed that *IGF1* was negatively regulated by different miRNAs at different differentiation time points (Figure 7B), indicating that the finely regulated miRNA network has an important influence on the spatiotemporal expression pattern of *IGF1*, which may regulate the process of muscle differentiation. In addition, we also analyzed the relationship between other key genes and their potential regulatory miRNAs during differentiation. The results showed that *Myf5* was negatively regulated by miR-137-3p, *Myh7* was co-regulated by miR-760-3p and miR-296-3p, *Myh8* was mainly inhibited by miR-335-3p, and *Mef2c* was the target of multiple miRNAs’ coordinated regulation (Figure S6).

These data combined indicate that miRNA plays a fine regulatory role in the proliferation and differentiation of C2C12 cells by forming a complex regulatory network with target genes, providing new insights into the molecular mechanism of muscle development.

## 4. Discussion

This study revealed the dynamic expression patterns of mRNA and miRNA and their regulatory networks during proliferation and differentiation of C2C12 myoblasts through systematic transcriptome analysis. Our data show that gene expression during myogenesis has significant temporal specificity and is precisely coordinated by complex transcriptional and post-transcriptional regulatory mechanisms.

During the proliferation stage, we observed a coordinated expression pattern of muscle progenitor cell-related transcription factors (such as *Pax7*) and cell cycle regulators, which is consistent with previous studies reporting the role of *Pax7* in maintaining the proliferation capacity of muscle stem cells [34]. In particular, in the early proliferation stage (0–12 h), we identified 339 significantly upregulated genes, which are mainly involved in DNA replication and cell cycle progression, which is consistent with previous studies on transcriptional activation after muscle stem cells exit the quiescent phase [35].

The role of miRNA in myogenesis is increasingly recognized [36]. This study found that miR-486 and miR-351 may have regulatory functions in the early stage of proliferation, which deserves further exploration. Previous studies have shown that miR-486 has multiple functions in muscle development and regeneration, especially affecting the muscle regeneration process by regulating the PTEN/AKT signaling pathway [37]. In addition, it has been reported that miR-486 affects the proliferation of sheep skeletal muscle satellite cells by regulating gene expression in the PI3K-Akt pathway [38]. It is worth noting that miRNA may have tissue-specific regulatory effects in different physiological contexts. For example, studies have found that the expression level of miR-351-5p is reduced in brain samples from mouse models with long-term chronic conditions (CCR). Therefore, this miRNA may play a key role in neuroprotection by regulating related pathways such as neurogenesis and might represent a potential therapeutic target for neurodegenerative diseases and aging-related diseases [39]. At the same time, studies have confirmed that miR-486a-5p is continuously dysregulated during the formation of choroidal neovascularization (CNV), and in vitro experiments have further demonstrated that it has a significant effect on the viability of mouse microglia, suggesting that it has potential application value in the treatment of CNV-related complications [40]. Other studies have shown that elevated IgE promotes cardiac fibrosis by inhibiting miR-486a-5p [41], while miR-486a-5p plays a protective role in ischemic reperfusion of the heart by inhibiting PDCD4 [42]. Our study further showed that miR-351-5p and miR-486a-5p may synergistically regulate the expression of proliferation-related genes in the early stage of myoblast proliferation.

During the differentiation stage, the temporal expression of muscle-specific transcription factors (*Myod1*, *Myog*) and myotube fusion factors (*Mymk*, *Mymx*) is highly consistent with the morphological changes of myotube formation. This observation supports the classic myogenic regulatory cascade model, in which *Myod1* activates *Myog* expression, which in turn synergistically drives the expression of muscle structural proteins and myotube fusion-related genes [43]. Through WGCNA analysis, we further identified key gene modules at each stage of differentiation, which are functionally enriched in signaling pathways such as MAPK and PI3K-Akt, consistent with the role of these pathways in promoting muscle differentiation [44]. Our study found that miR-335-3p and miR-760-3p may affect the differentiation process by regulating multiple key differentiation genes. The literature reports that miR-335-3p improves type II diabetes by regulating IGF-1-mediated macrophage polarization [45]. In addition, studies have confirmed that miR-335-3p is significantly upregulated after myoblast differentiation and promotes the differentiation process of myoblasts [46]. Our data further indicate that miR-335-3p may play an important regulatory role by targeting and regulating multiple key differentiation genes such as *IGF1*, *Mef2c*, and *Myh8*. Regarding miR-760-3p, studies have shown that it can activate the NF-kB pathway in cerebral ischemia by regulating Map3k8 [47] and can inhibit ferroptosis by targeting CHAC1 in neurons [48]. Although the role of miR-760-3p in myogenesis is still unclear, our study found for the first time that it may play an important role in myogenic differentiation by targeting key genes such as *Myh7*, *IGF1*, and *Fxyd1*.

Particularly striking, we found that *IGF1* might be regulated by a complex miRNA network during differentiation. *IGF1* is a key regulator of muscle growth and regeneration, activating the AKT/mTOR signaling pathway to promote protein synthesis and myofiber hypertrophy [49]. Our data suggest that *IGF1* might be dynamically regulated by different miRNAs at different differentiation stages, indicating the existence of precise regulatory mechanisms with temporal specificity. This finding is consistent with the emerging view that miRNAs act as “fine-tuners” in myogenesis, that is, miRNAs generally do not completely repress target gene expression, but regulate it to specific levels [17].

In addition, we identified a group of consistently upregulated genes (*Dio2*, *IGF1*, *Lum*, *Fxyd1*, *Pappa2*, and *Amy1*) that may represent new markers or regulators of muscle differentiation. Among them, *Dio2* (type 2 deiodinase) plays a key role in thyroid hormone activation and energy metabolism, and recent studies have shown its importance in muscle differentiation [50]. Similarly, the extracellular matrix protein Lum (leucine proteoglycan) may be involved in regulating the muscle microenvironment [51].

The limitation of this study is that it is based on in vitro cell models, and the biological functions of the identified regulators and signaling pathways need to be further verified by in vivo experiments in the future. In addition, the miRNA–mRNA pairing prediction process itself faces multiple challenges: (1) computational prediction methods may produce false positive targets, leading to over-interpretation of potential regulatory relationships; (2) existing prediction algorithms mainly rely on seed region complementarity analysis, which may ignore non-canonical targeting mechanisms; and (3) the complexity of the intracellular microenvironment and the dynamic regulation of competitive endogenous RNA networks may significantly affect actual molecular interactions. Although we have revealed a large number of potential miRNA–mRNA regulatory pairs through bioinformatics analysis, the direct regulatory relationships of these predicted results still need to be rigorously verified in subsequent studies through experimental methods to establish a more complete regulatory network model.

## 5. Conclusions

This study revealed the expression dynamics of mRNA and miRNA and their regulatory networks during the proliferation and differentiation of C2C12 myoblasts through systematic transcriptomic analysis. We identified key gene modules and potential miRNA regulatory axes in the proliferation and differentiation stages and found that *IGF1* expression was continuously upregulated during differentiation, which might be controlled by many specific miRNAs. This provides new insights into the molecular mechanisms of muscle development.

## Data Availability

The data presented in this study are available upon request from the corresponding author.

## Supplementary Materials

The following supporting information can be downloaded at: https://www.mdpi.com/article/10.3390/biology14050574/s1. Figure S1: Cell number statistics in the same field of view at the corresponding time points in Figure 1A; Figure S2: (A) Venn diagram of differentially expressed genes at adjacent time points of differentiation. (B) RNA-seq analysis of *Amy1*, *Dio2*, *Fxyd1*, *Igf1*, *Lum*, and *Pappa2* gene expression during C2C12 cell differentiation (*n* = 3). Bars represent mean ± SEM. * *p* < 0.05, ** *p* < 0.01, *** *p* < 0.001; Figure S3: GS and MM analysis between candidate module genes and time groups. (A) GS and MM analysis between brown module genes and proliferation for 12 h. (B) GS and MM analysis between candidate module genes and differentiation time groups; Figure S4: Histogram of the results of the cross-analysis between DEGs and DE-miRNAs predicted target genes; Figure S5: GO and KEGG enrichment analysis of intersection genes between DEGs and predicted target genes of DE-miRNAs. (A) Proliferation. (B) Differentiation; Figure S6: Interaction network of key genes with negatively regulated miRNAs during differentiation.
