# Supplementary material for: Integrated miRNA–mRNA Profiling of C2C12 Myoblasts Indicates Regulatory Interactions Involved in Proliferation and Differentiation
Source: Biology (Basel). 2025 May 20;14(5):574. doi: 10.3390/biology14050574 (PMC12108691; doi:10.3390/biology14050574)
Supplement: Supplementary file 1 [file biology-14-00574-s001.zip › biology-3631768-supplementary.pdf]

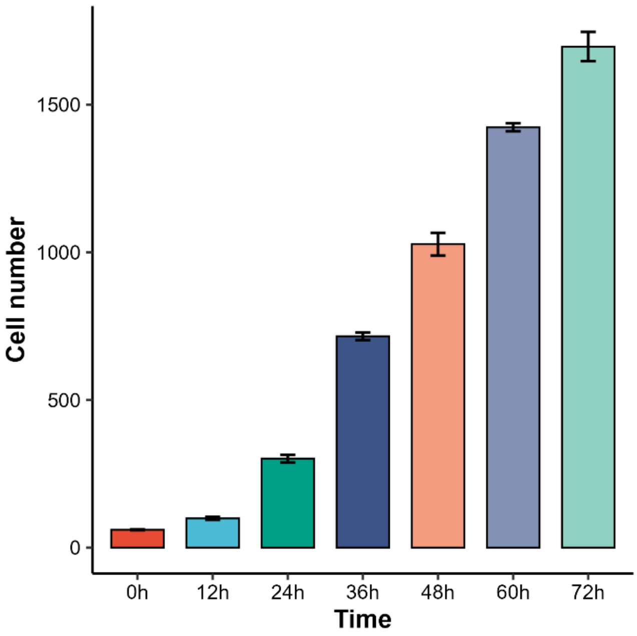

Figure S1: Cell number statistics in the same field of view at the corresponding time points in Figure 1A.

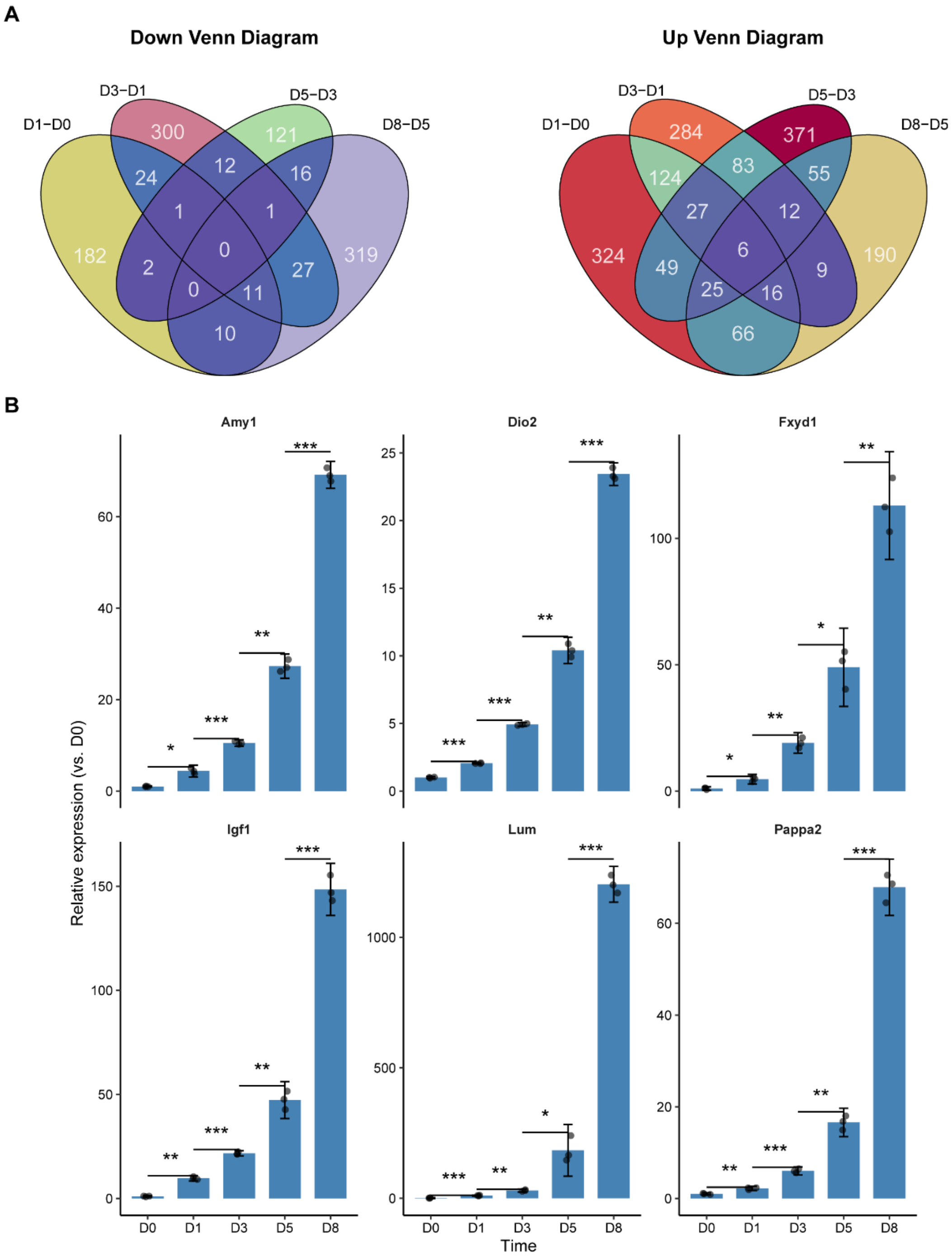

Figure S2. (A) Venn diagram of differentially expressed genes at adjacent time points of differentiation. (B) RNA-seq analysis of *Amy1*, *Dio2*, *Fxyd1*, *Igf1*, *Lum*, and *Pappa2* gene expression during C2C12 cell differentiation (n = 3). Bars represent mean ± SEM. *$p < 0.05$, **$p < 0.01$,***$p < 0.001$ (Student's t test).

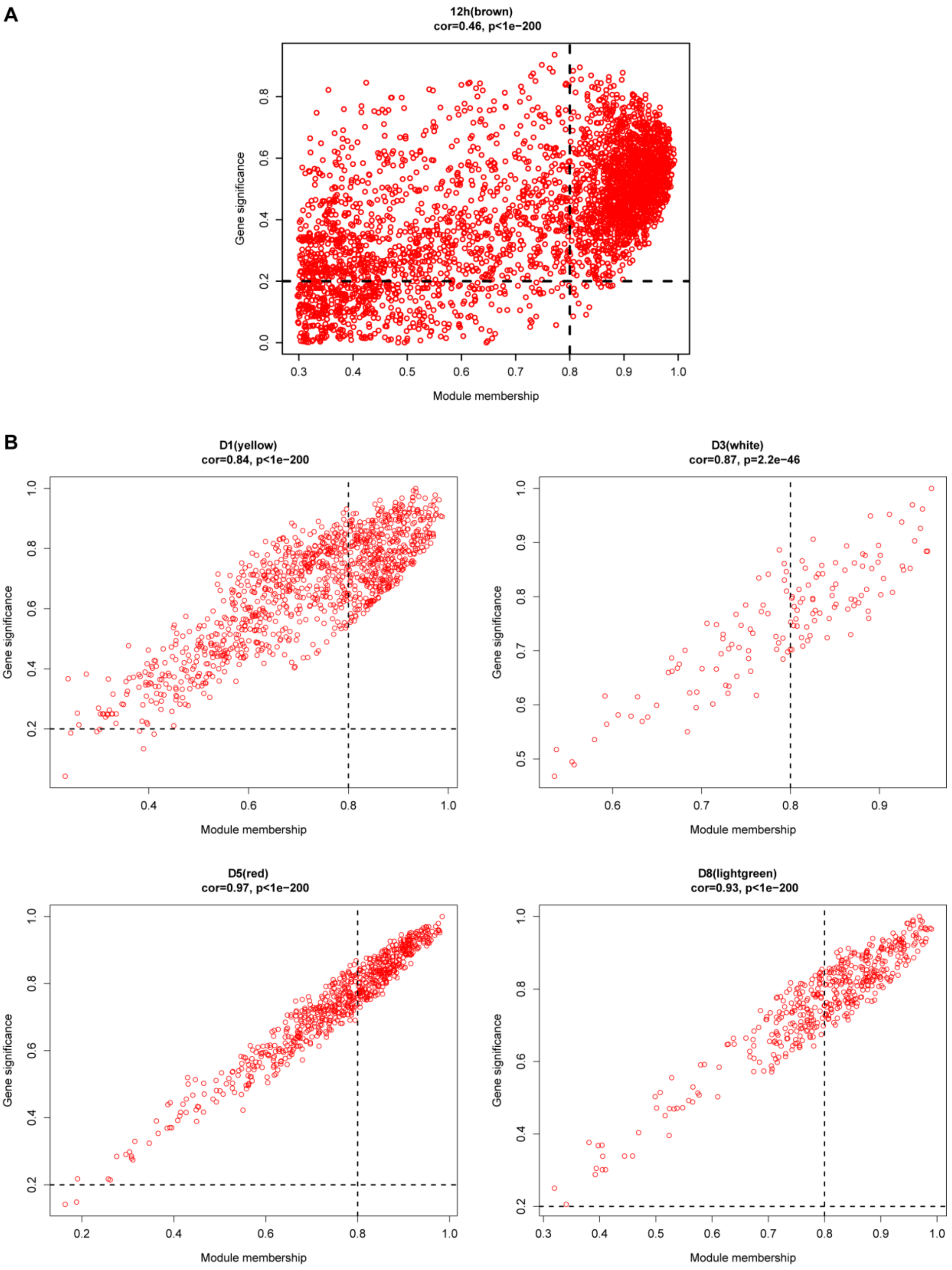

Figure S3. GS and MM analysis between candidate module genes and time groups.

(A) GS and MM analysis between brown module genes and proliferation for 12 h.

(B) GS and MM analysis between candidate module genes and differentiation time groups.

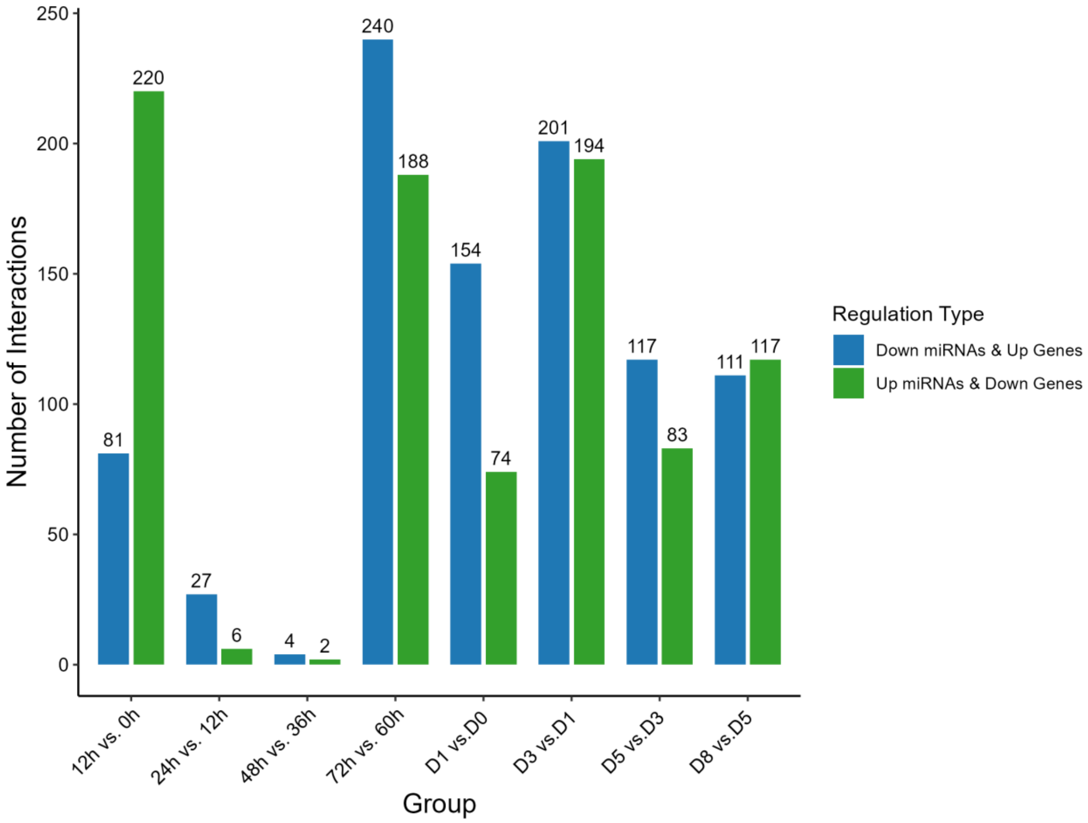

Figure S4. Histogram of the results of the cross-analysis between DEGs and DE-miRNAs predicted target genes.

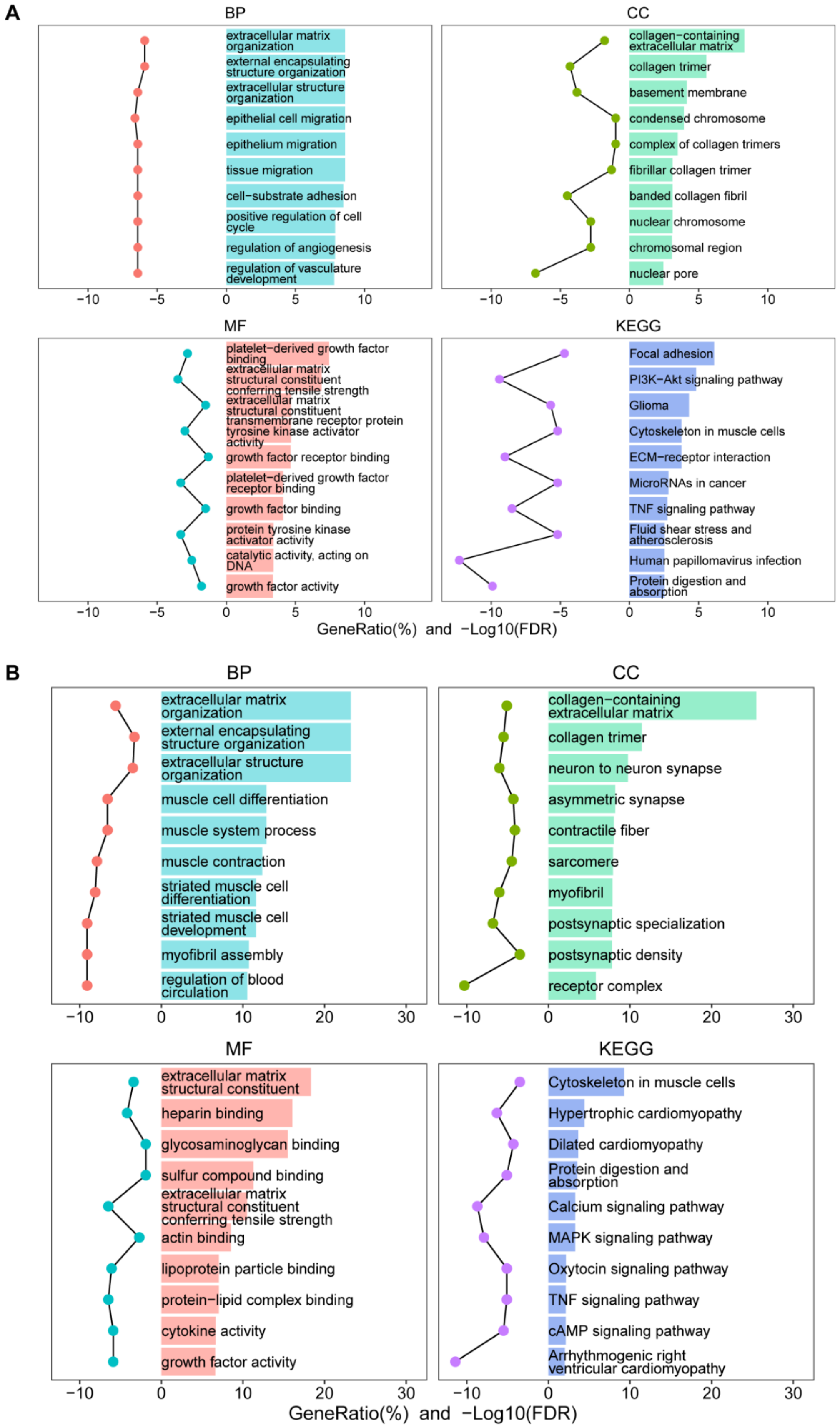

Figure S5. GO and KEGG enrichment analysis of intersection genes between DEGs and predicted target genes of DE-miRNAs. (A) Proliferation. (B) Differentiation.

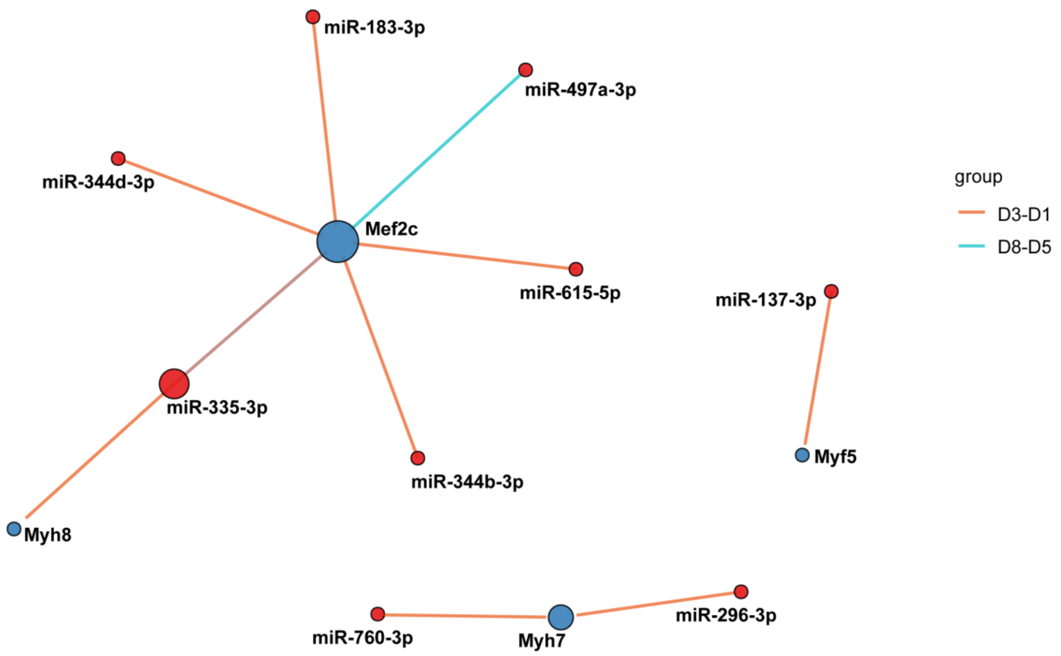

Figure S6. Interaction network of key genes with negatively regulated miRNAs during differentiation.
